# Combined blockade of MEK and PI3KCA as an effective antitumor strategy in *HER2* gene amplified human colorectal cancer models

**DOI:** 10.1186/s13046-019-1230-z

**Published:** 2019-06-04

**Authors:** Valentina Belli, Nunzia Matrone, Stefania Napolitano, Giorgia Migliardi, Francesca Cottino, Andrea Bertotti, Livio Trusolino, Erika Martinelli, Floriana Morgillo, Davide Ciardiello, Vincenzo De Falco, Emilio Francesco Giunta, Umberto Bracale, Fortunato Ciardiello, Teresa Troiani

**Affiliations:** 1 Medical Oncology, Department of Precision Medicine, Università degli Studi della Campania “Luigi Vanvitelli”, Via S. Pansini 5, 80131 Naples, Italy; 2 Division of Cancer Medicine, Department of Gastrointestinal Medical Oncology, The University of Texas MD Anderson Cancer Center, Houston, USA; 3 Department of Oncology, University of Torino, 10060 Candiolo, Turin, Italy; 4 Candiolo Cancer Institute – FPO IRCCS, 10060 Candiolo, Turin, Italy; 5 Department of Endocrinology, Gastroenterology and Endoscopic Surgery, Università di Napoli Federico II, 80131 Naples, Italy

**Keywords:** Colorectal cancer, HER2-amplified cancer, MEK and PI3KCA inhibitors; xenografts; patient-derived xenografts

## Abstract

**Background:**

Targeting the epidermal growth factor receptor (EGFR) either alone or in combination with chemotherapy is an effective treatment for patients with *RAS* wild-type metastatic colorectal cancer (mCRC). However, only a small percentage of mCRC patients receive clinical benefits from anti-EGFR therapies, due to the development of resistance mechanisms. In this regard, HER2 has emerged as an actionable target in the treatment of mCRC patients with resistance to anti-EGFR therapy.

**Methods:**

We have used SW48 and LIM1215 human colon cancer cell lines, quadruple wild-type for *KRAS, NRAS, BRAF* and *PI3KCA* genes, and their *HER2*–amplified (LIM1215-HER2 and SW48-HER2) derived cells to perform in vitro and in vivo studies in order to identify novel therapeutic strategies in *HER2* gene amplified human colorectal cancer.

**Results:**

LIM1215-HER2 and SW48-HER2 cells showed over-expression and activation of the HER family receptors and concomitant intracellular downstream signaling including the pro-survival PI3KCA/AKT and the mitogenic RAS/RAF/MEK/MAPK pathways. *HER2*-amplified cells were treated with several agents including anti-EGFR antibodies (cetuximab, SYM004 and MM151); anti-HER2 (trastuzumab, pertuzumab and lapatinib) inhibitors; anti-HER3 (duligotuzumab) inhibitors; and MEK and PI3KCA inhibitors, such as refametinib and pictilisib, as single agents and in combination. Subsequently, different in vivo experiments have been performed. MEK plus PI3KCA inhibitors treatment determined the best antitumor activity. These results were validated in vivo in *HER2*-amplified patient derived tumor xenografts from three metastatic colorectal cancer patients.

**Conclusions:**

These results suggest that combined therapy with MEK and PI3KCA inhibitors could represent a novel and effective treatment option for *HER2*-amplified colorectal cancer.

**Electronic supplementary material:**

The online version of this article (10.1186/s13046-019-1230-z) contains supplementary material, which is available to authorized users.

## Background

In the last decade, the development of targeted anti-cancer therapies has revolutionized the treatment of metastatic colorectal cancer (mCRC) patients. In particular, monoclonal antibodies (mAbs) targeting the epidermal growth factor receptor (EGFR) like cetuximab and panitumumab were the first targeted agents to enter the clinical setting improving the survival of mCRC patients [1, 2]. However, efficacy in anunselected patient population was limited, whereas the identification of biomarkers has led to significantly improved patient selection [3]. In particular, mutations in *KRAS* and *NRAS* genes are found to predict resistance to anti-EGFR targeted therapies and are used in clinical practice to guide treatment decision [4]. Furthermore, at least one third of mCRC patients with *RAS* wild type tumors receiving first-line chemotherapy in combination with anti-EGFR mAbs fail to have a therapeutic response. These results indicate that additional genetic alterations in genes implicated in the EGFR signaling network can be involved in the primary resistance [5–8]. In fact, deregulation of other effectors of the EGFR signaling cascade, such as mutations in *BRAF* or *PIK3CA* genes, loss of *PTEN* expression, and amplification of *KRAS* may affect primary response to EGFR blockade [9–12]. Despite the implementation of biomarkers in clinical practice, patients who initially respond to anti-EGFR therapies almost invariably develop secondary resistance through several mechanisms. The most common molecular mechanisms that are responsible for acquired resistance are genetic alterations of *KRAS*, *NRAS* and *BRAF* genes [6, 13]. In the absence of alteration in *RAS* or its immediate downstream effectors, other mechanisms have been involved in the activation of the EGFR pathway. Genetic aberrations in receptor tyrosine kinase (RTK), such as HER2 and MET, have been shown to bypass EGFR signaling and activate the MAPK cascade and, therefore, to confer acquired resistance to anti-EGFR therapies [14–16]. In particular, *HER2* amplification has been suggested as both an intrinsic as well as an acquired mechanism of resistance [17]. One explanation could be that pre-exiting infrequent *HER2*-amplificated clones might be expanded under the selective pressure of anti-EGFR therapy, leading to disease progression. In this regard, *HER2* amplification was found in 5% of mCRC patients with *RAS* wild type tumors and seem to be associated with resistance to anti-EGFR therapy [18, 19]. In a large cohort of 85 patient-derived colorectal cancer xenografts, Bertotti and colleagues identified *HER2* gene amplification in some xenografts, which were resistant to cetuximab and did not harbour mutations in *KRAS*, *NRAS* or *BRAF* genes [17, 20, 21]. Moreover, patient-derived mCRC xenografts with *HER2* amplification were treated with various HER2-targeted therapies, alone or in combination. In these preclinical models of human colorectal cancer, the combination of an anti-HER2 antibody (pertuzumab or trastuzumab) and an HER2 tyrosine kinase inhibitor (TKI) (lapatinib) induced pronounced tumor shrinkage [17]. These preclinical results were the proof of concept for clinical trials targeting *HER2* genetic alterations in mCRC patients [22]. The phase II HERACLES-A trial of dual HER2-targeted therapy (trastuzumab plus lapatinib) in patients with *KRAS* wild-type, *HER2*-positive mCRC who were refractory to standard-of-care treatments, including cetuximab or panitumumab was conducted [23]. Of the 27 patients evaluated for efficacy, eight patients (30%) achieved an overall objective response, meeting the primary endpoint of the trial. None of the patients enrolled who were evaluable for response to anti-EGFR therapy had achieved an objective response with either cetuximab or panitumumab. Based on these results, several clinical trials have been conducted exploiting *HER2* as a target for mCRC and also case reports of patients with *HER2*-positive mCRC who have achieved substantial clinical benefit with targeted anti-HER2 therapy have recently been published [19, 24, 25]. However, 40–50% of patients treated within the HERACLES-A trial did not achieve partial response or prolonged stable disease despite *HER2* gene amplification [23, 26]. Notably, even in patients initially responding, acquired resistance occurred in almost all cases [23]. Understanding the mechanisms of resistance to HER2 blockade is a priority to develop more effective and additional options for therapy in this disease setting.

In order to elucidate the possible mechanism(s) of resistance to anti-HER2 treatments, in this study we have used LIM1215 and SW48 human colon cancer cell lines and their *HER2*-amplified derivatives (LIM1215-HER2 and SW48-HER2) to perform in vitro and in vivo studies using different xenograft models in order to identify novel therapeutic options [17, 23, 26]. Furthermore, human mCRC patient derived tumor xenografts with *HER2* gene amplification were used to further validate the potential efficacy of these therapeutic strategies.

## Methods

### Drugs

5-Fluorouracil, oxaliplatin and irinotecan were obtained from the pharmacy of the University of Campania “Luigi Vanvitelli”. Cetuximab, panitumumab, SYM004, MM151, trastuzumab, pertuzumab and duligotuzumab antibodies were kindly provided by Merck, Amgen, Symphogen, Merrimack Pharmaceuticals, Roche and Genentech, respectively. Refametinib, a selective MEK 1/2 inhibitor was kindly provided by Bayer Italy; Pictilisib, a PI3Kα inhibitor and lapatinib were purchased from Selleckchem.

### Human cancer cell lines

The human LIM1215 and SW48 colon cancer cell lines were purchased from the American Type Culture Collection (ATCC). Cells were grown in RPMI 1460 medium supplemented with 10% fetal bovine serum and 1% penicillin/streptomycin and maintained in a humidified controlled atmosphere at 37 °C.

### Colony formation assay

LIM1215-HER2 and SW48-HER2 cells were treated with different concentrations of trastuzumab, pertuzumab, lapatinib, cetuximab, SYM004, MM-151, duligotuzumab, refametinib and pictilisib alone and in combination (range: 0,05–10 μg/ml-μM), for 96 h. After 14 days, cells were fixed with 4% paraformaldehyde and stained with crystal violet. Finally, the plates were inspected by microscopy, photographed and the colonies area was calculated by ImageJ plugins [27]. Results represent the median of three separate experiments, each performed in triplicate.

### Western blot analysis

LIM1215-HER2 and SW48-HER2 cells were treated with refametinib and pictilisib alone and in combination for 24 h. Equal amounts of total proteins were incubated with following primary polyclonal antibodies: EGFR and phospho-EGFR, HER2 and phospho-HER2, HER3 and phospho-HER3, HER4 and phospho-HER4, AKT and phospho-AKT, MEK1/2 and phospho-MEK1/2, p44/42MAPK and phospho-p44/42 purchased from Cell Signaling. Monoclonal anti- α-tubulin antibody was provided by Sigma-Aldrich. After incubation with secondary anti-goat antibody, the membranes were developed using an enhanced chemi-luminescence (ECL) detection system (BioRad). As concerning the in vivo experiments, tumor samples were homogenized as previously described [28]. Indicated proteins were probed with the same antibodies, as described above.

### Tumor xenografts in nude mice

Four- to six-week old female balb/c athymic (nu+/nu+) mice were purchased from Envigo Laboratories. Three different in vivo experiments were performed. Briefly for the first experiment, mice were injected subcutaneously in the right flank with LIM1215-HER2 and SW48-HER2 cells and were randomly assigned to one of the following groups (10 mice/group). Group 1: vehicle administrated intraperitoneally (i.p.); group 2: refametinib administrated by oral gavage (o.g.) every day (25 mg/kg); group 3: pictilisib administrated by o.g. every day (75 mg/kg); group 4: combined treatment of refametinib (25 mg/kg) and pictilisib (75 mg/kg). Treatments were continued for 4 weeks. In the second in vivo experiment, groups of 90 mice injected in the right flank with LIM1215-HER2 and SW48-HER2 cells were treated with oxaliplatin (10 mg/kg) once every 2 weeks (i.p) plus trastuzumab (10 mg/kg) twice a week (i.p). At the end of 4 weeks treatment (induction treatment), mice were randomized into one of the following nine groups: Group 1: Veichle control; group 2: Pictilisib administrated by o.g. every day (75 mg/kg); group 3: refametinib administrated by o.g. every day (25 mg/kg); group 4: trastuzumab (10 mg/kg) twice a week i.p.; group 5: refametinib plus trastuzumab; group 6: pictilisib plus trastuzumab; group 7: pictilisib plus refametinib; group 8: lapatinib plus trastuzumab; group 9: the triplet combination of pictilisib, refametinib and trastuzumab. All drugs in the combined treatment are used at same concentration as single agent. Treatments continue for 8 weeks (maintenance treatment) and afterwards animals were followed for additional 16 weeks (follow-up period). At the end of maintenance treatment, one animal *per* group was sacrificed and tumor sample were collected for western blot analysis. In the third in vivo experiment, mice injected in the right flank with LIM1215-HER2 and SW48-HER2 cells were divided into four groups. Group 1: Vehicle control; group 2: refametinib (25 mg/kg) plus pictilisib (75 mg/kg) for 26 weeks; group 3: lapatinib (30 mg/kg) plus trastuzumab (10 mg/kg) for 26 weeks; group 4: lapatinib (30 mg/kg) plus trastuzumab (10 mg/kg) until progression. At the time of progression to lapatinib plus trastuzumab, mice were treated in second-line with combination of refametinib (25 mg/kg) and pictilisib (75 mg/kg). Tumor volume was measured using the formula π/6 × larger diameter x (smaller diameter)^2^. For monitoring tumor responses to therapy, we measured volumetric changes and used an arbitrary classification method modified from clinical research methods as previously reported [28].

### Tumor specimen collection and annotation

Tumor and matched normal samples were obtained from patients treated by liver metastasectomy at the Candiolo Cancer Institute (Torino, Italy), Mauriziano Umberto I, and San Giovanni Battista (Torino). All patients provided informed consent.

### Patient derived xenograft (PDX) models and in vivo treatments

Tumor implantation and expansion were performed in 6-week-old NOD/SCID mice as previously described [17, 29]. Mice were randomized and treated with the following agents: trastuzumab; refametinib; pictilisib. Tumor size was measured twice a week. Results were considered interpretable when a minimum of 4 mice *per* treatment group reached the pre-specified endpoints (at least 3 weeks on therapy or development of tumors with volumes larger than 1500 mm^3^) [30].

### Statistical analysis

Statistical analyses of the in vitro data were performed using a one-way analysis of variance (ANOVA). Quantitative data were reported as mean ± standard deviation (SD). Results were compared by ANOVA.

## Results

### In vitro generation and characterization of *HER2*-amplified human colorectal cancer cell lines

The human CRC cell lines SW48 and LIM1215 harbor the wild type (WT) *KRAS, NRAS, BRAF*, and *PIK3CA* genes and are considerate an optimal preclinical model of EGFR therapeutic blockade. Since both cell lines have no or little expression of HER2 protein, they were transfected with *HER2* gene in order to generate their *HER2*-amplified derivatives cells (SW48-HER2 and LIM1215-HER2). Transfection of *HER2* gene results in cell stably overexpressing the HER2 protein (Additional file 1: Figure S1).To characterize the phenotype of these cells, we have studied the HER2 protein function on cell mobility. As illustrated in Additional file 1: Figure S2 A-B, parental SW48 and LIM1215 cells exhibited significant migratory capability. On the contrary their *HER2*-amplified derivatives cells demonstrated no ability in migration. These findings suggest that the amplification of *HER2* gene is responsible of epithelial-mesenchymal-transition (EMT) loss. To further support this hypothesis, the expressions of epithelial- and mesenchymal-related proteins were measured. As depicted in Additional file 1: Figure S2 C-D, protein expression of different epithelial and mesenchymal markers changes between parental and *HER2*-amplified derivatives cells. In particular, the expressions of vimentin and slug, two mesenchymal markers, were decreased or suppressed in SW48-HER2 and LIM1215-HER2 cells, as compared to parental cells, respectively. On the contrary, E-cadherin, a common epithelial marker, strongly increased in both *HER2*-amplified derivatives cells as compared to parental cells. Collectively, these results suggest that colon cancer cell lines with *HER2* amplification have lost an invasive behavior and have acquired an epithelial phenotype. In this scenario, we have performed additional western blot analyses to evaluate differential expression of other HER family receptors and their downstream effectors among parental cell lines SW48 and LIM1215 and their derived, *HER2*-amplified cancer cells. Western blot analysis showed an increased expression and phosphorylation of EGFR, HER3 and HER4 in *HER2*-amplified derivatives, that could lead to a complex intracellular signaling which includes the activation of the pro-survival PI3KCA/AKT pathway and of the mitogenic MAPK pathway [31, 32]. Activation of MAPK, MEK and AKT with an increase in their phosphorylated forms was observed in both *HER2*-amplified cells as compared to their parental counterpart (Additional file 1: Figure S3 A-B and Additional file 2: Table S1).

It has been demonstrated that HER2 is the only member of the HER family receptors that has no ligand and, therefore, it is activated mostly via hetero-dimerization with other HER family receptors [24, 33]. Therefore, we have analyzed whether the activation of AKT and MAPK effectors in *HER2*-amplified colon cancer cells could be due to the interaction of HER2 with other HER family receptors. For this purpose, SW48, SW48-HER2, LIM1215, LIM1215-HER2 protein extracts were immune-precipitated with a specific anti-HER2 antibody and then assayed by western blotting with a specific anti-HER3, anti-HER4 and anti-EGFR antibodies. As shown in Additional file 1: Figure S3-C, HER2 immuno-precipitated together with HER3 and EGFR forming HER2/HER3 and HER2/EGFR complexes only in SW48-HER2 and LIM1215-HER2 cells, but not in SW48 and LIM1215 cells. No immune-complexs were formed between HER2 and HER4 (data not shown). These data suggested the activation of HER2 signaling and this effect leads to activation of the pro-survival PI3K/AKT pathway and the mitogenic MAPK pathway through HER2/HER3 and HER2/EGFR heterodimerization.

### Sensitivity to chemotherapeutic agents and to anti-EGFR monoclonal antibodiesin parental and in *HER2*-amplified human colon cancer cell lines

We first tested in vitro the activity of different chemotherapeutic agents in both parental and *HER2*–amplified colon cancer cell lines. All cancer cells we treated with 5-fluorouracil, oxaliplatin and irinotecan for 96 h. As shown in Additional file 1: Figure S4-A, there was no differential sensitivity to chemotherapies among all cancer cells. We next evaluated the sensitivity to the cell growth inhibiting effects of different anti-EGFR mAbs, including cetuximab, panitumumab, SYM004, and MM-151. As shown in Additional file 1: Figure S4-B, there was a differential sensitivity to anti-EGFR mAbs-induced cell growth inhibition. In fact, parental SW48 and LIM1215 cancer cells were significantly sensitive to cetuximab, panitumumab, SYM004 and MM-151 antibodies inducing growth inhibition, as expected being “quadruple wild type” for *KRAS, BRAF, NRAS* and *PIK3CA* genes. On the contrary, *HER2*-amplified colon cancer cells were resistant to all anti-EGFR inhibitors, expanding and confirming the results from previous studies regarding the role of *HER2* amplification in either primary and acquired resistance to EGFR targeted therapies [17, 34, 35].

### Effects of anti-HER2 inhibitors alone and/or in combination with anti-EGFR and anti-HER3 monoclonal antibodies on *HER2*-amplified human colon cancer cells

**Fig. 1:**
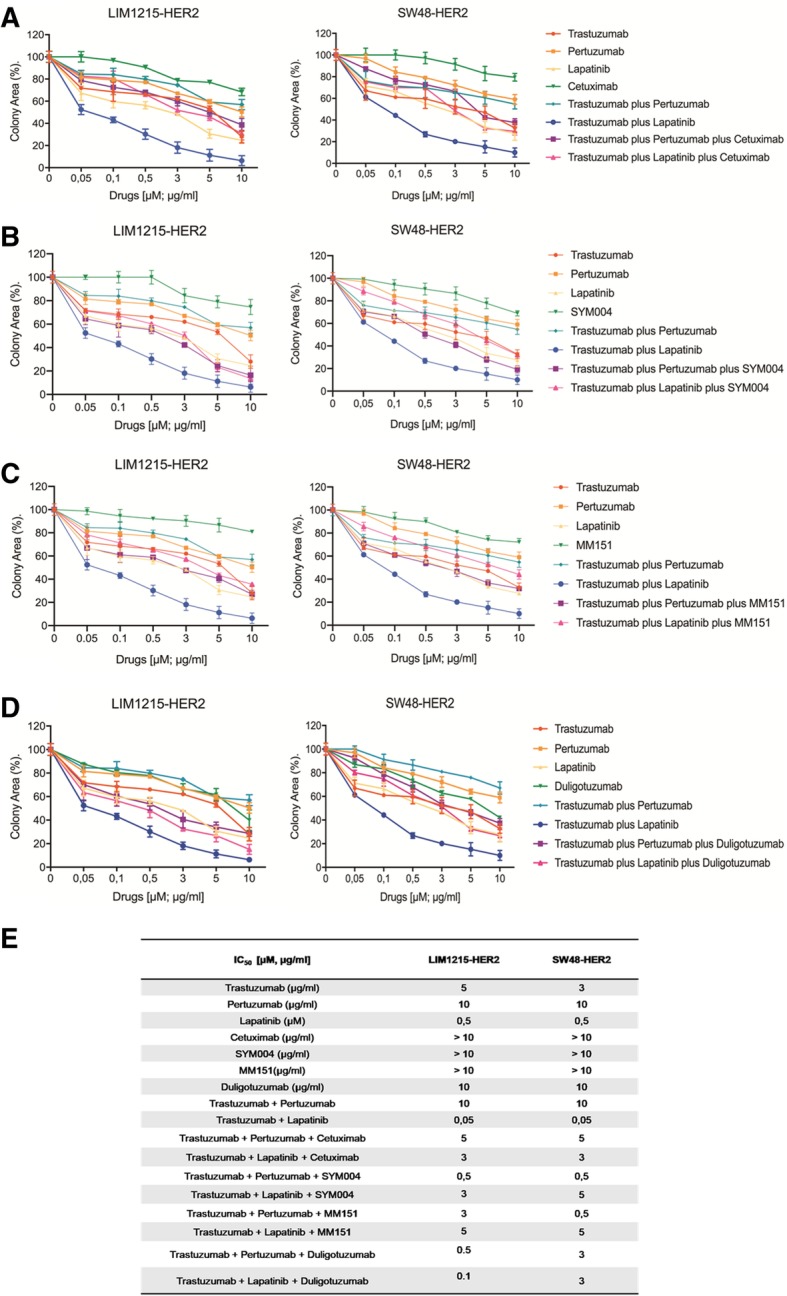
Effects of dual block of anti-HER2 inhibitors alone and/or in combination with anti-EGFR and anti-HER3 mAbs on *HER2*-amplified (SW48-HER2 and LIM1215-HER2) human colon cancer cells. **a**-**e** Cells sensitivity to different drugs was evaluated by clonogenic assay in LIM1215-HER2 and SW48-HER2 cells. Data were represented as cell colony area calculated by ImageJ software after staining with crystal violet, as described in Materials and Methods. **e** Table with IC_50_ value for each drug, used as single agent or in combination

In previous studies, it has been demonstrated that *HER2* amplification represents not only a biomarker of resistance to EGFR inhibition, but also a positive predictor of response to HER2 targeting agents [17]. In fact, mCRC patient derived xenografts with *HER2* amplification were sensitive to HER2-blockade with trastuzumab in combination with lapatinib, but not to either agent alone. In HER2-amplified colon cancer cells monotherapy with either HER2 tyrosine kinase inhibitor or anti-HER2 antibodies was almost ineffective, whereas only the combination of a monoclonal antibody, trastuzumab, and a tyrosine kinase inhibitor, lapatinib, induced a significant anti-proliferative activity (Fig. 1a-e). Our data showed that, whereas pertuzumab, trastuzumab and lapatinib treatments had no or little effects as single agents on cell growth in LIM1215-HER2 and SW48-HER2 cells, a dual blockade of HER2 with trastuzumab and lapatinib had the most anti-proliferative effect compared to other treatments (Fig. 1 a-e). Moreover, this effect was not ameliorated by the addition of the other two anti-HER2 mAbs, as trastuzumab and pertuzumab. Furthermore, in *HER2*-amplified LIM1215-HER2 and SW48-HER2 colon cancer cells HER2 activation was accompanied by the interaction of HER2 with both EGFR and HER3, respectively, by forming HER2/EGFR and HER2/HER3 complexes. Therefore, we investigated whether a dual blockade of HER2 by using lapatinib and trastuzumab, that is considered the most active therapeutic strategy, in combination with anti-EGFR or anti-HER3 inhibitors could be a more effective treatment in *HER2*-amplified cells. As depicted in Fig. 1 a-e, the addiction of anti-EGFR mAbs to lapatinib plus trastuzumab combined treatment antagonized the anti-proliferative activity of the dual anti-HER2 blocked. In particular, in LIM1215-HER2 cells the IC_50_ for the triplet combination with trastuzumab, lapatinib and cetuximab was 3 μg/ml, 60 times higher than the IC_50_ of dual anti-HER2 blockade. Similar results were obtained by using triplet combinations with the anti-HER3 antibody duligotuzumab (Fig. 1 a-e).

### Effects of refametinib and pictilisib on cell proliferation and on EGFR-dependent intracellular pathways in parental SW48 and LIM1215 and in *HER2*-amplifiedhuman colon cancer cell lines

**Fig. 2:**
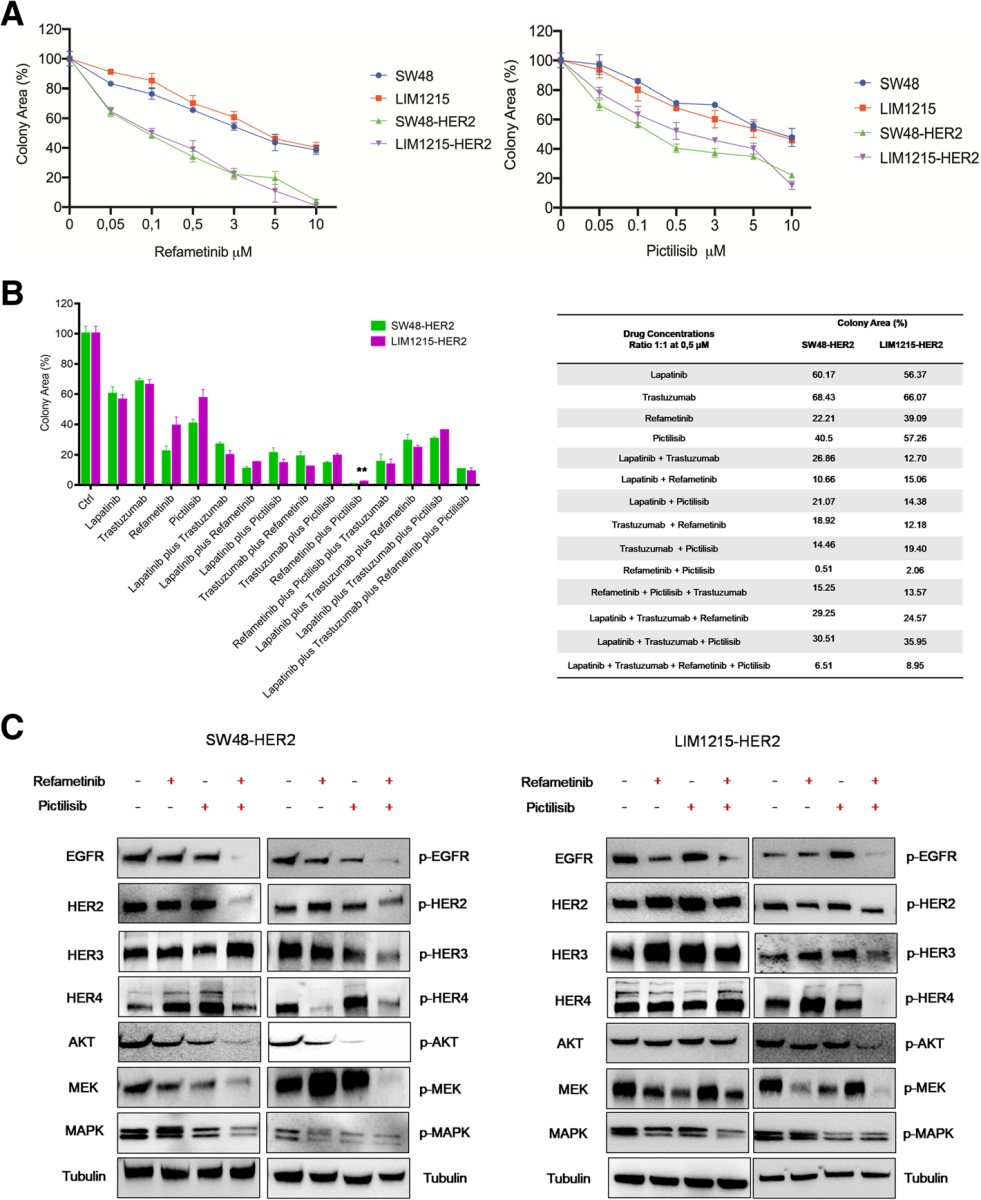
Effects of refametinib and pictilisib alone and in combination on colonies formation and on EGFR-dependent intracellular signaling in parental SW48 and LIM1215 human colon cancer cell lines and in their *HER2*-amplified derivatives (SW48-HER2 and LIM1215-HER2). **a** Both parental and HER2-amplified derivatives cells were treated with different concentrations of refametinib and pictilisib as single agents (range, 0.05 to 10 μM), for 96 h and evaluated byclonogenic assay. Data were represented as cell colony area percentage calculated by ImageJ software after staining with crystal violet, as described in Materials and Methods. **b** SW48-HER2 and LIM1215-HER2 cell lines were treated with lapatinib, trastuzumab, refametinib and pictilisib at 0.5 μM, as single agent and in their possible combinations (**p < 0.01 compared to other treatments). Colony area percentage was presented in the Table. **c** SW48-HER2 and LIM1215-HER2 cells were treated with refametinib and pictilisib (0.5 μM) alone and in combination for 24 h. Cell protein extracts were subjected to immunoblotting with the indicate antibodies, as described in Materials and Methods. α-Tubulin was used as the loading control. Results represent the mean of three separate experiments, each performed in duplicate

Given the observed concomitant activation of MAPK and PI3KCA-driven intracellular signals in *HER2*-amplified colon cancer cells, we next evaluated the cell growth inhibiting effects of a selective MEK1/2 inhibitor, refametinib, and of a selective PI3Kα inhibitor, pictilisib, as single agents or in combination. First, to evaluate the sensitivity to MEK and/or PI3KCA inhibitors between parental and derived cells, SW48, LIM1215, SW48-HER2 and LIM1215-HER2 cell lines were treated with both drugs as a single agent. As shown in Fig. 2-a, although refametinib and pictilisib treatments caused a dose-dependent cell growth inhibition in all colon cancer cell lines, the *HER2*-amplified cells were more sensitive to both drugs, as single agents, compared to parental cells at all concentrations tested. Next, we evaluated whether the treatment of refametinib in combination with pictilisib could induce a more effective anti-proliferative effect as compared to single treatments in *HER2*-amplifed colon cancer cell lines. The combined treatment with refametinib and pictilisib induced significant growth inhibition in both SW48-HER2 and LIM1215-HER2 cells (Fig. 2-b). To further evaluate if anti-HER2 blockade with trastuzumab or lapatinib could increase the anti-proliferative effects of refametinib plus pictilisib, SW48-HER2 and LIM1215-HER2 cells were treated with trastuzumab, lapatinib, refametinib and pictilisib, in different combinations. As depicted in Fig. 2-b, treatments with anti-HER2 agents, trastuzumab and lapatinib alone and in combination, had no effect when added to the refametinib plus pictilisib combined treatment. These results suggest that combination of refametinib plus pictilisib is more effective than lapatinib plus trastuzumab combined treatment and that the addition of anti-HER2 drugs to MEK and PI3KCA inhibitors does not significantly increase cell growth inhibition in these *HER2*-amplified colon cancer models. To better understand whether the anti-tumor activity obtained by the combined treatment with MEK and PI3KCA inhibitor was due to a more effective inhibition of key intracellular signals, EGFR downstream signaling pathways were evaluated. SW48-*HER2* and LIM1215-*HER2* cells were treated with refametinib, pictilisib and/or their combination. The combined treatment with refametinib plus pictilisib substantially inhibited the expression of EGFR, HER2, HER4 and their downstream effectors, such as AKT, MEK, and MAPK in their total forms and consequently the combination blocked their activation compared with single-agent treatments after 24 h of incubation. The concomitant block of PI3KCA/AKT and MAPK pathways could generate a negative feedback loop that led to the reduction in expression and activation of proteins. However, further investigation is needed to elucidate in detail these molecular mechanisms (Fig. 2c).

### Effects of refametinib and pictilisib on *HER2*-amplified human cancer xenografts

**Fig. 3:**
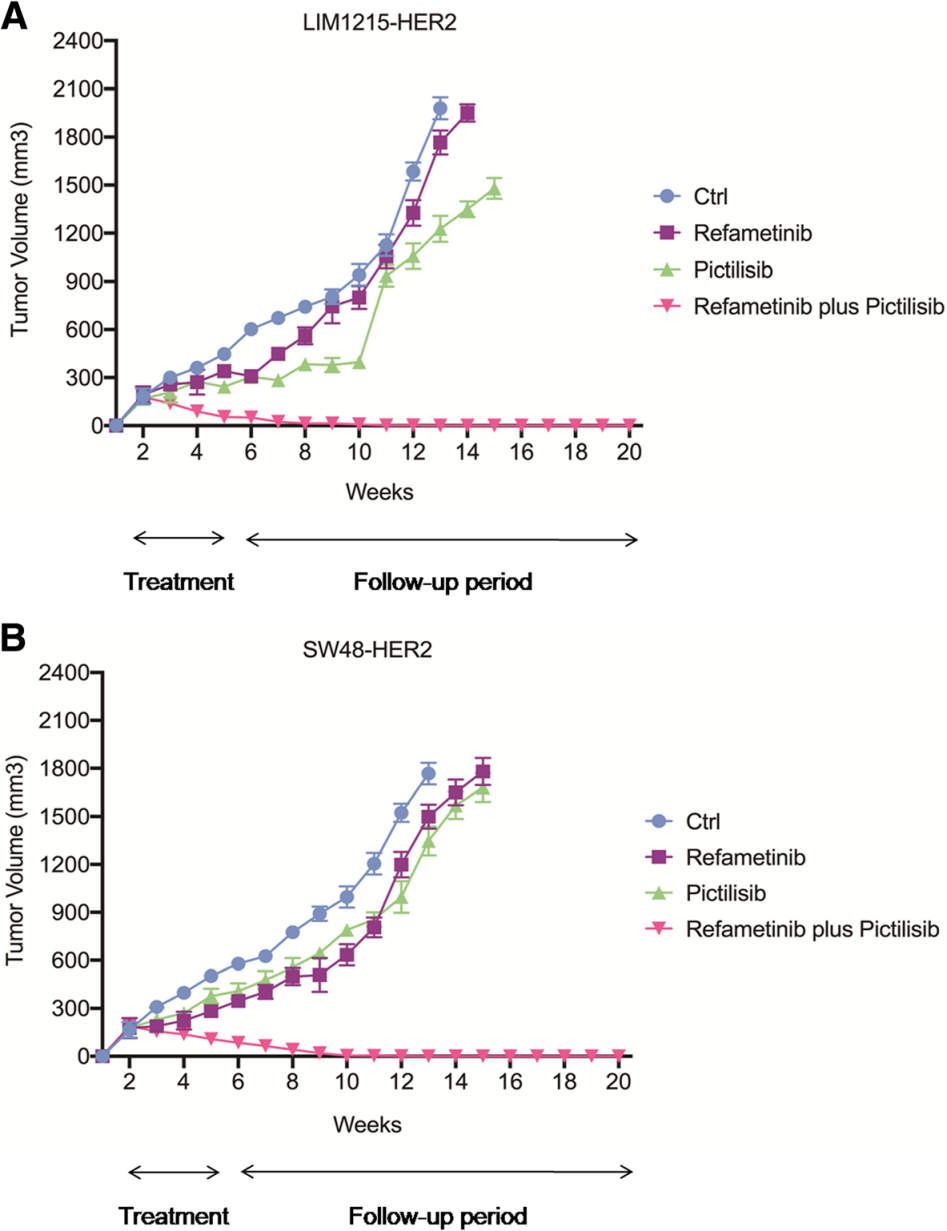
Effects of refametinib and pictilisib on LIM1215-HER2 and SW48-HER2 tumor xenografts. Mice were injected subcutaneously in the right flank with (**a**) LIM1215-HER2 and (**b**) SW48-HER2 cell lines, as described in Materials and Methods. After 2 weeks (average tumor size 200 mm^3^) mice were treated with refametinib (25 mg/kg every day, by o.g.) and pictilisib (75 mg/Kg every day, by o.g.), as single agents and in combination, for 4 weeks. Thereafter, tumor growth was followed without any further treatment until the 20th week. Data are means ± SD of ten mice in each group

Subsequently, we investigated the in vivo antitumor activity of refametinib and pictilisib. Mice injected with LIM1215-HER2 and SW48-HER2 cells were randomly assigned to receive vehicle, refametinib (25 mg/kg), pictilisib (75 mg/Kg) or their combination for 4 weeks. As shown in Fig. 3, treatment with refametinib had little or no effect on tumor growth in both tumor xenografts. Similar results were obtained in the groups treated with pictilisib alone. On the contrary, the combined treatment suppressed almost completely LIM1215-HER2 and SW48-HER2 tumor growth at the end of the 4 weeks of therapy (Fig. 3 a-b). This complete suppression of tumor growth in the combined treatment group was long lasting up to week 20.

### Antitumor efficacy of oxaliplatin plus trastuzumab followed by maintenance treatment with different inhibitors alone or in combination with trastuzumab in *HER2*-amplified human cancer xenografts

**Fig. 4:**
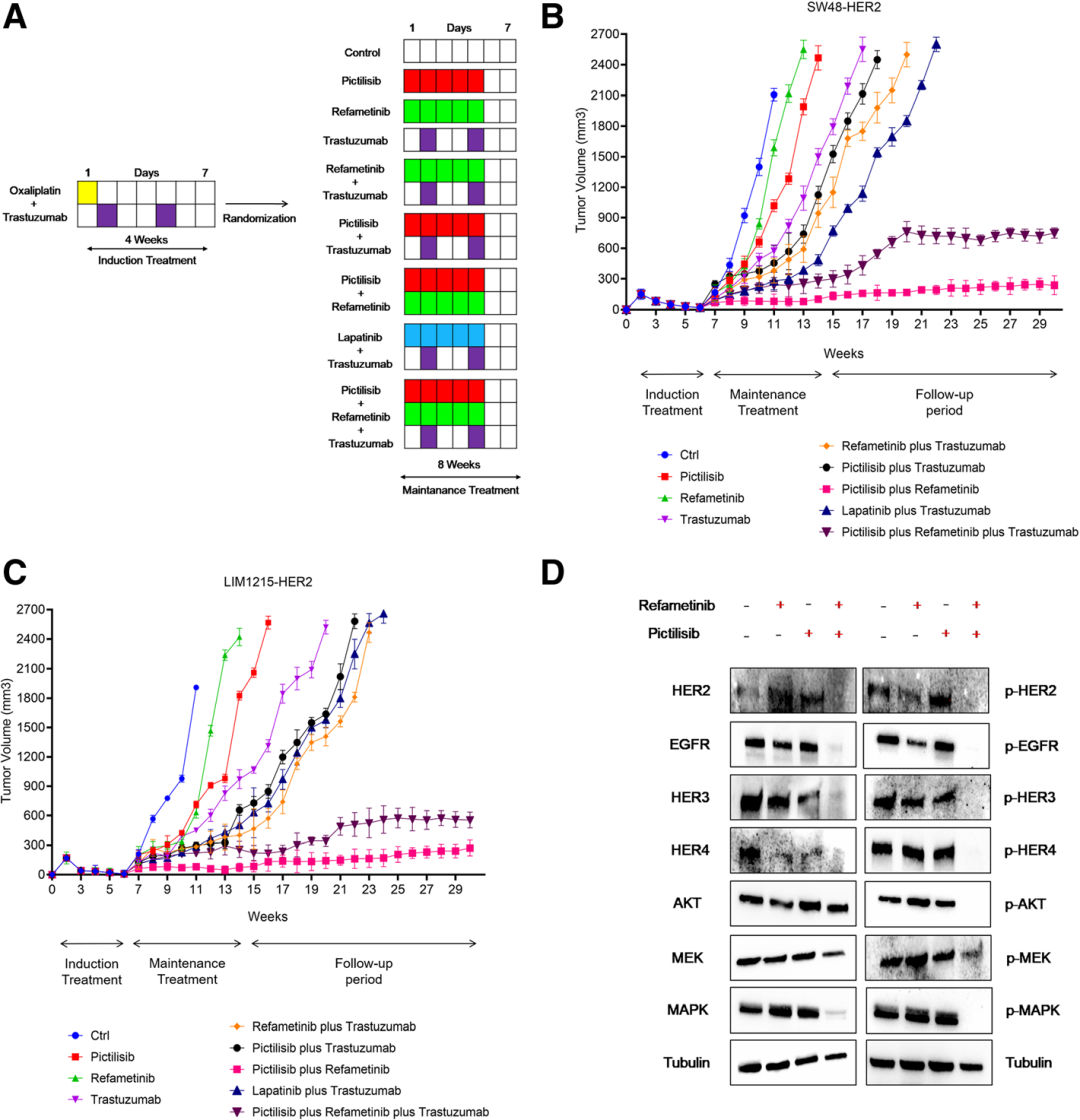
Effect of maintenance treatment with different kinase inhibitors alone or in combination with trastuzumab, after induction therapy with oxaliplatin plus trastuzumab in *HER2*-amplified colon cancer xenograft models. *HER2*-amplified colon cancer cells (SW48-HER2 and LIM1215-HER2) were injected into the right flank of nude mice. After 2 weeks of subcutaneous injection, mice were treated with oxaliplatin (10 mg/kg once every 2 weeks, i.p.) in combination with trastuzumab (10 mg/kg twice a week, i.p.) for 4 weeks (induction treatment). Afterward, mice were randomized into nine groups and treated for 8 weeks (maintenance treatment). Ctrl: control; pictilisib (75 mg/Kg) and refametinib (25 mg/kg) were administrated every day for 5 days by o.g, as single agents and in different combinations; trastuzumab (10 mg/kg twice a week, by i.p.) and lapatinib (30 mg/kg every day, by o.g.) as single agents and in different combinations. **a** Treatment scheme. Red boxes: pictilisib treatment days; green boxes: refametinib treatment days; violet boxes: trastuzumab treatment days; light blue boxes: lapatinib treatment days. **b**–**c** Antitumor activity of maintenance treatment in SW48-HER2 and LIM1215-HER2 tumor-bearing mice. The indicated cancer cell lines were grown as subcutaneous tumor xenografts in nude mice and treated with different drugs as indicated above. The mean data are present. Tumor growth curves were calculated on the basis of three times a week tumor measurements during the treatment period and after 16 weeks of observation after termination of therapy. **d** Analysis of EGFR-dependent intracellular signaling by western blotting in LIM1215-HER2 colorectal cancer xenograft. At the end of maintenance treatment, 1 mouse *per* group treated with refamentinib, pictilisib or with their combination was sacrificed. Tumor samples were collected, and total cell protein extracts were subjected to immunoblotting with the indicated antibodies, as described in materials and methods. Anti-tubulin antibody was used for normalization of protein extract content

In a subsequent in vivo experiment, two groups of 90 nude mice were injected subcutaneously with each *HER2*-amplified cancer cells and were treated for 4 weeks with the combination of oxaliplatin plus trastuzumab. At the end of this treatment, mice were randomized into nine groups and treated for 8 weeks with different inhibitors, including refametinib, pictilisib, lapatinib and trastuzumab alone or in combination (maintenance period) (Fig. 4) a-c. After the maintenance period, mice were followed for an additional 16 weeks. At the end of the maintenance treatment (week 14), among the single-agent treatments, the group treated with trastuzumab showed the greatest tumor growth inhibition in both xenograft models with a mean tumor volume of 971 and 1500 mm^3^, respectively (Fig. 4 a-c and Additional file 2: Table S2). In fact, the growth rate of tumors treated with pictilisib and refametinib as single agent was similar to those treated with vehicle regardless of cell lines injected reaching the maximum allowed tumor size of 2000 mm^3^ (Fig. 4 a-c and Additional file 2: Table S2). In the combined treatment groups, although the addition to refametinib and/or pictilisib to trastuzumab caused a strong antitumor activity in both xenograft models, the most effective antitumor activity was observed with the combined treatment of refametinib plus pictilisib. In particular, this combination caused an almost complete suppression of tumor growth in LIM1215-HER2 and SW48-HER2 tumor xenografts with a mean tumor volume of 75 and 103 mm^3^, respectively. Moreover, only in this treatment group mice with no evidence of tumors were observed (Fig. 4 a-c and Additional file 2: Table S2). Mice were followed for additional 16 weeks (follow-up period) from the end of the treatment to evaluate which maintenance strategy could determine a more sustained and prolonged tumor growth control. In the combined trastuzumab plus refametinib or pictilisib groups, tumors started to regrowth immediately after the cessation of treatment. Indeed, 3 to 5 weeks after cessation of treatment the tumor growth rate in these combined treatment groups was comparable to the tumor growth rate in the trastuzumab group for both *HER2*-amplified cancer models. On the contrary, the anti-tumor activity of the combined refametinib plus pictilisib treatment was still maintained within 13 to 16 weeks after the cessation of therapy. In particular, in this combined treatment group almost half of the mice were still alive at the end of the follow-up period (Fig. 4a-c and Additional file 2: Table S2). Moreover, in order to evaluate the mechanism(s) by which the combined treatment with refametinib plus pictilisib was able to induce a significant and long lasting antitumor activity, one mouse for each group treated with refametinib, pictilisib or with their combination was sacrificed at the end of the maintenance treatment. As shown in Fig. 4-d, the combined treatment with refametinib plus pictilisib was the only treatment able to substantially inhibit the expression and activation of HER receptors family and their downstream effectors in their total forms at the end of the maintenance treatment.

**Fig. 5:**
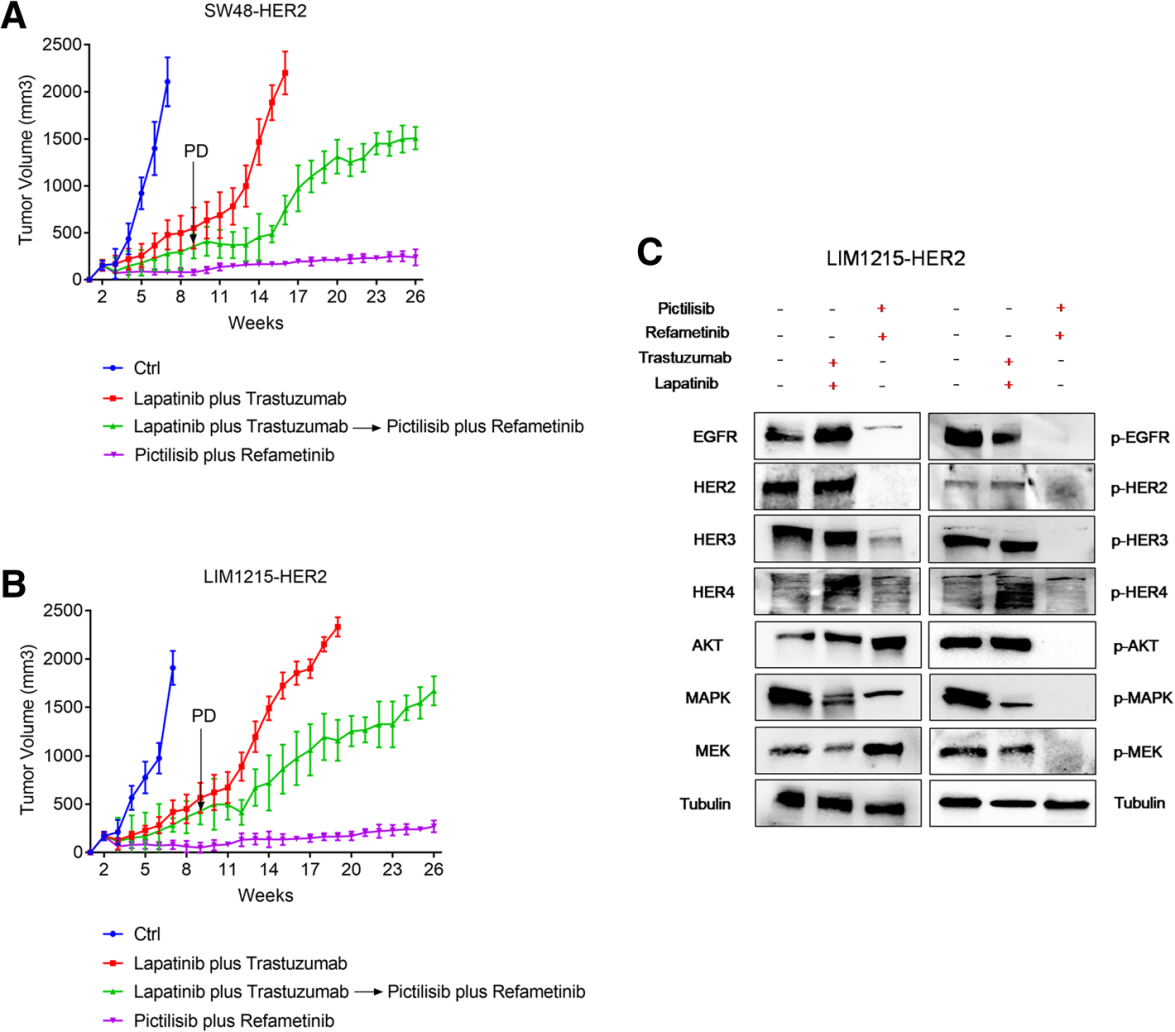
Effect of refametinib and pictilisib combined treatment after tumor progression due to lapatinib plus trastuzumab therapy in *HER2*-amplified colon cancer xenograft models. **a**-**b** SW48-HER2 and LIM1215-HER2 were injected into right flank of nude mice. After 2 weeks from subcutaneous injection, mice were divided in four groups and treated for 26 weeks. Group 1: ctrl. Group 2: lapatinib (30 mg/kg every day, o.g.) and trastuzumab (10 mg/kg twice a week, i.p.) were administrated in combination. Group 3: refametinib (25 mg/kg every day, o.g.) and pictilisib (75 mg/Kg every day, o.g.) were administrated in combination. Group 4: mice were treated with combination of lapatinib (30 mg/kg every day, o.g.) and trastuzumab (10 mg/kg twice a week, i.p.) until progression (defined as > 30% increment of tumor volume from baseline). After tumor progression of group 4, mice were treated in second-line with combination of refametinib and pictilisib. **c** Tumor samples were collected and total protein extracts were subjected to immunoblotting with all antibodies, as described in materials and methods section. Anti-tubulin antibody was used for normalization of protein extract content

To further dissect the effects of refametinib plus pictilisib combined treatment on *HER2*-amplified xenograft models, LIM1215-HER2 and SW48-HER2 cells were injected subcutaneously into nude mice and were treated with veichle, lapatinib plus trastuzumab or pictilisib plus refametinib until tumors increased growth (Fig. 5 a-b). When tumors were growing despite treatment with lapatinib plus trastuzumab, treatment with refametinib plus pictilisib was started. As shown in Fig. 5, treatment with refametinib plus pictilisib provided further antitumor activity. However, this effect was transient. In fact, after 5 weeks of this therapy, tumors started to regrowth reaching the maximum allowed tumor size of 2000 mm^3^ at week 26. On the contrary, the group of mice treated with refametinib plus pictilisib from the beginning of the experiment experienced the best antitumor efficacy with no evidence of tumor progression up to week 26 (Fig. 5 a-b). Moreover, the combined treatment with refametinib plus pictilisib as initial therapy substantially inhibited phosphorylation of EGFR, HER2, HER3, HER4 and of downstream signaling pathways such as MEK, MAPK, and AKT, as measured by western blotting at the end of the treatment period (Fig. 5-c). Collectively, these data support the hypothesis that in *HER2*-amplified colon cancer xenografts treatment with refametinib plus pictilisib is more effective as initial therapy in order to better control the onset of cancer cell resistance mechanisms, that are more frequent and develop earlier with HER2 blockade.

### Effect of refametinib and pictilisib combined treatment with or without trastuzumab in *HER2*-amplified metastatic colorectal cancer patient derived tumor xenografts

**Fig. 6:**
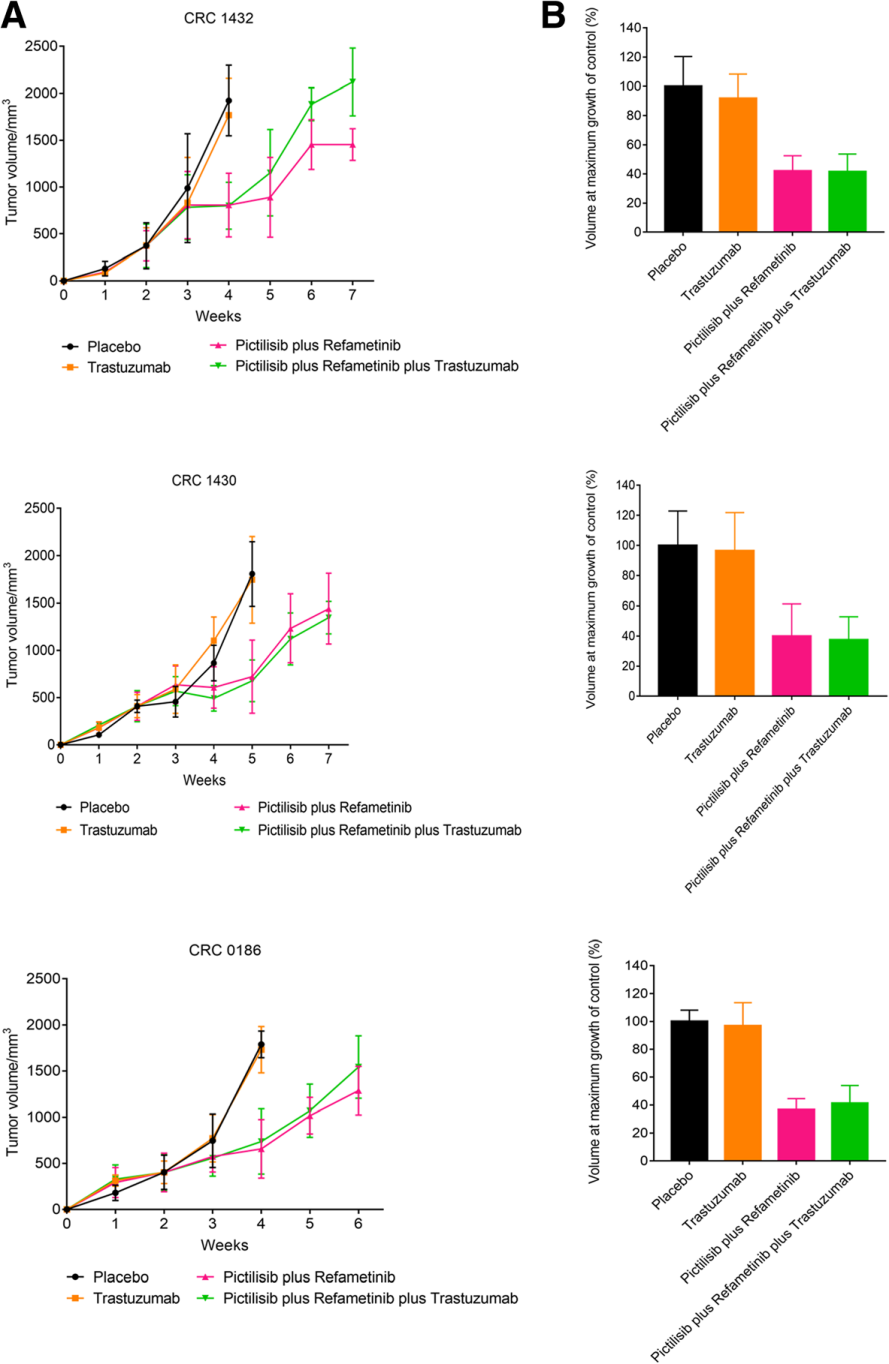
Effect of refametinib and pictilisib combined treatment with or without trastuzumab in *HER2*-amplified metastatic colorectal cancer patient derived tumor xenografts. Tumor samples with HER2 amplification derived from three different patients were directly implanted and expanded in mice. **a** Representative tumor growth curves of tumors in xenopatients derived from quadruple-negative HER2-amplified cases CRC 1432, CRC 130, CRC 0186 treated with the indicated modalities for 5 weeks: trastuzumab 10 mg/kg i.p., twice weekly; refametinib 25 mg/kg by o.g., daily; pictilisib 75 mg/kg by o.g., daily. **b** Histogram represents the tumor volume of each treatment compared to the maximum growth of the control at the fourth week

One way to proceed with efficient, high-fidelity drug development at the stage of in vivo validation while minimizing the effects of uncharacterized tumor heterogeneity is to perform preclinical population-based studies by using human cancer specimens directly transplanted into mice (“xenopatients”) [17]. For this reason, to further evaluate the antitumor efficacy of refametinib plus pictilisib as a therapeutic strategy for *HER2*-amplified colon cancer we used patients derived tumor xenografts (PDTXs), by selecting three representative cases (CRC 1432, CRC 1430 and CRC 0186) of *HER2*-amplified tumors from mCRC patients [17]. For each experiment, mice were divided into four groups and treated for 5 weeks with vehicle, trastuzumab, refametinib plus pictilisib or the triple combination of refametinib, pictilisib and trastuzumab (Fig. 6 a-b). The combination treatment with refametinib plus pictilisib and triple combination of refametinib, pictilisib and trastuzumab were highly effective in suppressing tumor growth, as compared to either the vehicle-treated controls or the trastuzumab single agent treatment group. Moreover, the combined treatment with refametinib plus pictilisib exhibited an even greater antitumor activity as compared to the triple combination.

## Discussion

In the past decade, the development of targeted therapies has provided new options for the personalized management of patients with advanced solid tumors [1]. In particular, CRC represents a heterogeneous group of diseases with different sets of genetic events, accompanying immune response, and influences of exogenous factors, providing a challenge for personalized therapeutic approaches [1]. The mAbs directed against the EGFR, such as cetuximab and panitumumab, are currently approved for the treatment of patients with mCRC [1–5]. Despite significant progress in strategies for cancer treatment, these therapies have improved patient responses and their use is limited by the presence of pre-existing intrinsic resistance mechanisms or by the ability of cancer cells to acquire resistance [6–10].

One of major mechanism of acquired resistance to anti-EGFR mAbs is the activation of growth-factor signaling pathways by upregulation of alternative and compensatory signaling cascades through receptors other than EGFR [14–16]. In particular, *HER2* amplification has been suggested as a mechanism of resistance [14, 17]. One explanation could be that pre-existing infrequent *HER2*-amplified clones might be expanded under the selective pressure of anti-EGFR therapy, leading to disease progression [20]. In this regard, *HER2* amplification is more likely to confer acquired anti-EGFR therapy resistance. *HER2* amplification was found in 5% of mCRC patients harboring no mutation in *KRAS, NRAS, BRAF, PI3KCA* genes [17–20] and together with “xenopatients” preclinical data provided the rational for clinical studies with HER2-targeting therapies after failure of anti-EGFR treatments [17, 23, 24]. The HERACLES-A clinical trial reported clinical meaningful responses to dual inhibitor HER2-directed therapy in a subset of *HER2*-amplified mCRC patients, whose disease was refractory to chemotherapy and anti-EGFR antibodies [23, 36].

Nevertheless, the mechanisms of resistance to HER2-directed and EGFR-directed therapies are still unclear. An improved understanding of the molecular characteristics of *HER2*-amplified CRC models and their potential mechanisms of resistance to HER2-directed therapy may influence the direction of future research on targeted therapies and inform future therapeutic decision-making.With this aim, starting from quadruple wild-type human CRC cells for *KRAS*, *NRAS*, *BRAF* and *PI3KCA* genes (LIM1215 and SW48), we have generated *HER2*-amplified (LIM1215-HER2 and SW48-HER2) cells. In the current study, we report the up-regulation of HER family receptors and over-expression of several markers involved in RAS/RAF/MAPK and PI3KCA-AKT pathways in *HER2*-amplified cells compared to parental colon cancer cells. Interestingly, these oncogenic pathways were activated by the interaction between HER2/HER3 and HER2/EGFR, consequent to the heterodimerization of these receptors. In agreement with these findings, several studies have demonstrated that the acquired resistance to anti-EGFR mAbs was associated with the increased activation not only of HER2, but also of HER3 and/or other alternative receptor tyrosine kinases with consequent activation of EGFR-independent intracellular downstream pathways [37, 38]. Based on these data, in order to inhibit the compensatory feedback effect due to activation of downstream signaling pathways, we have tested as therapeutic strategy the combination of two selective MEK and PI3KCA inhibitors, refametinib and pictilisib, respectively, in *HER2*-amplified colon cancer cells (LIM1215-HER2 and SW48-HER2). The combined treatment with refametinib plus pictilisib determined a strong antitumor activity both in vitro and in vivo, providing the rationale for the further clinical development of this combination. In particular, in nude mice bearing LIM1215-HER2 or SW48-HER2 CRC tumor xenografts, the combined treatment with refametinib plus pictilisib caused complete tumor regression that lasted up to 20 weeks of follow up after the end of treatment. These results suggest that the concomitant blockade of two key intracellular signaling hubs that could be involved in the development of cancer cell resistance to anti-HER2 inhibitors might be a possible strategy to delay or prevent its onset. Moreover, to extend the validation and the potential clinical relevance of these findings, we have performed an additional in vivo experiment. We treated mice first with a combination of oxaliplatin plus trastuzumab for 4 weeks as a strategy to treat *HER2*-amplified mCRC patients [23]. Before tumor started to regrowth and eventually possible resistance mechanisms to targeted agents would occur, mice were randomized to different maintenance treatments with several kinase inhibitors, such as refametinib, pictilisib, lapatinib or trastuzumab alone or in combination. The combined treatment with refametinib plus pictilisib again was the best therapeutic strategy also in this experimental maintenance therapy setting. Moreover, these findings have been further validated in three *HER2*-amplified mCRC patient derived xenografts, which could better resemble the complexity of a human mCRC, since these models have been demonstrated as a better surrogate of human cancer and may represent a valid experimental tool to overcome the limitations of in vitro models by faithfully recapitulating the histological and functional heterogeneity observed in primary tumor samples. The combined treatment with refametinib plus pictilisib exhibited a significant antitumor activity, that was accompanied by a sustained tumor growth inhibition in all three *HER2*-amplified mCRC patient derived xenografts.

Collectively, these data suggest that the dual and combined inhibition of MEK and PI3KCA significantly inhibit tumor growth in several different models of *HER2*-amplified CRC both in vitro and in vivo. In this respect, early clinical trials have evaluated the combined inhibition of MEK and PI3KCA signaling pathways in different tumor types [39–42]. In particular, although the effect of PI3KCA pathway activation as a mechanism of resistance to HER2-directed therapy in CRC has not been directly investigated, activating mutations of PI3KCA and decreased expression of PTEN have been identified as potential mechanisms of resistance to trastuzumab and lapatinib in breast cancer [43, 44]. A study using human breast cancer cell lines and mouse tumor xenografts has shown that activating mutations of PIK3CA and/or decreased expression of PTEN could be responsible of resistance to lapatinib and that this resistance is reversible by double blockade of PI3KCA and mTOR [45].

The ongoing Personalized Oncogenomics Group (POG) trial (NCT02155621) has undertaken complete molecular characterization of a total of 60 patients with mCRC, two of which showed high-level of *HER2* amplification. With the aim of better understanding mechanisms of resistance to HER2-directed and EGFR-directed therapies, Owen et al. reported the complete molecular characterization of these two cases of *HER2*-amplified mCRC. Their findings included increased expression of *MUC1* and *MET*, decreased expression of *PTEN*, and an activating mutation in *PIK3CA* [46]. These data support the hypothesis that a potentially promising alterative to overcome resistance mechanisms would be to apply a therapy in the upfront setting in order to suppress and ideally eradicate pre-existing resistant clones while they still are present in a low frequency subpopulation.

## Conclusions

In summary, the present study provides experimental evidence that the combined treatment with refametinib plus pictilisib could be a potential novel therapeutic strategy to treat *HER2*-amplified mCRC patients with the aim of improving the outcome of this aggressive disease subgroup which is mostly refractory to standard therapies.

## Additional files

Additional file 1: **Figure S1.** Expression and phosphorylation of HER2 in parental SW48 and LIM1215 human colon cancer cell lines and in their HER2-amplified derivatives (SW48-HER2 and LIM1215-HER2) cells (Additional file 3: Supplementary Methods). **Figure S2.** Phenotypic characterization of parental SW48 and LIM1215 human colon cancer cell lines and of their HER2-amplified derivatives (SW48-HER2 and LIM1215-HER2) cells. **Figure S3.** Expression and phosphorylation of HER family receptors and their downstream signaling pathways in parental SW48 and LIM1215 human colon cancer cell lines and in their HER2-amplified derivatives (SW48-HER2 and LIM1215-HER2) cells. **Figure S4.** Effects of chemotherapeutic agents and of anti-EGFR monoclonal antibodies on cell proliferation in parental SW48 and LIM1215 human colon cancer cell lines and in their HER2-amplified derivatives (SW48-HER2 and LIM1215-HER2) cells. (DOCX 5258 kb)

Additional file 2: **Table S1.** Evaluation of protein expression level in parental (SW48 and LIM1215) and in HER2-amplified human colon cancer cell lines. Legend: Negative symbol (-) no protein expression detected; Positive symbols: (+) expression and (++) over-expression levels of each protein detected. **Table S2.** Antitumor efficacy of oxaliplatin plus trastuzumab followed by maintenance treatment in human HER2-amplified colon cancer xenograft. (DOCX 613 kb)

Additional file 3: Supplementary Methods. (DOCX 18 kb)

## Abbreviations

Akt: Protein Kinase-B
CRC: colorectal cancer
EGFR: Epidermal growth factor receptor
EMT: epithelial- mesenchymal transition
HER family: Epidermal growth factor receptor family
HER2: Human epidermal growth factor receptor 2
HER3: Human epidermal growth factor receptor 3
HER4: Human epidermal growth factor receptor 4
HERACLES: HER2 Amplification for ColorectaL Cancer Enhanced Stratification
mAbs: monoclonal antibodies
MAPK: mitogen-activated protein kinase
mCRC: metastatic colorectal cancer
PDTX: patient derived tumor xenograft
PI3KCA: Phosphatidylinositol 4,5-bisphosphate 3-kinase
WT: wild-type
