# Supplementary material for: Combined blockade of MEK and PI3KCA as an effective antitumor strategy in HER2 gene amplified human colorectal cancer models
Source: J Exp Clin Cancer Res. 2019 Jun 4;38:236. doi: 10.1186/s13046-019-1230-z (PMC6549349; doi:10.1186/s13046-019-1230-z)

**Additional Figures and Legends**

**Figure S1. Expression and phosphorylation of HER2 in parental SW48 and LIM1215 human colon cancer cell lines and in their *HER2*-amplified derivatives (SW48-HER2 and LIM1215-HER2) cells.** The SW48 and LIM1215 colon cancer cells were transfected with HER2 plasmid, as described in Supplementary Materials and Methods. Western blot analysis of HER2 and phospho-HER2 in SW48, SW48-HER2, LIM1215 and LIM1215-HER2 cellswas performed as described in Supplementary Materials and Methods. α-Tubulin was used as aprotein loading control.

**Figure S2. Phenotypic characterization of parental SW48 and LIM1215 human colon cancer cell lines and of their *HER2*-amplified derivatives (SW48-HER2 and LIM1215-HER2) cells. (A-B) Migration Assay.** (A). Cell migration ability of SW48, LIM1215, SW48-HER2 and LIM1215-HER2 cells was tested by Transwell migration assay as described in Supplementary Materials and Methods. The Boyden chambers were stained by crystal violet and images were taken by microscope at 10X. (B) The number of cells migrated was measured by spectrophotometer (**p < 0.01). **(C-D) Expression of selected epithelialand mesenchymal-related proteins.** (C) Expression of EMT markers, such as E-cadherin, vimentin and slug, was evaluated by western blot analysis in SW48, LIM1215, SW48-HER2 and LIM1215-HER2 cells. α-Tubulin was used as aprotein loading control. (D) Immunofluorescence staining of EMT markers pictured by laser confocal microscopy with 40X objective in SW48, LIM1215, SW48-HER2 and LIM1215-HER2 cells. Scale bar, 10 µm.

**Figure S3. Expression and phosphorylation of HER family receptors and their downstream signaling pathways in parental SW48 and LIM1215 human colon cancer cell lines and in their *HER2*-amplified derivatives (SW48-HER2 and LIM1215-HER2) cells.** (A) Basal expression of HER family receptors and (B) their downstream effectors in SW48, LIM1215, SW48-HER2 and LIM1215-HER2 cells. α-Tubulin was used as a protein loading control. (C) Protein extraction of SW48-HER2, LIM1215-HER2 and parental cells were immune-precipitated with a specific anti-HER2 antibody and then immune-blotted with anti-HER3 and anti-EGFR antibodies, as described in Supplementary Materials and Methods.Input: cell lysate of SW48 and LIM1215 cells and their HER2-amplified derivatives; IP: immuno-precipitated proteins with HER2 antibody.

**Figure S4. Effects of chemotherapeutic agents and of anti-EGFR monoclonal antibodies on cell proliferation in parental SW48 and LIM1215 human colon cancer cell lines and in their *HER2*-amplified derivatives (SW48-HER2 and LIM1215-HER2) cells.** (A) Cells were treated with different concentrations of chemotherapeutic agents such as 5-fluorouracil, oxaliplatin and irinotecan (range, 0.05-25 μg/ml) for 96 hours and evaluated for proliferation by MTT assay. (B) Cells were treated with different concentrations of anti–EGFR monoclonal antibodies such as cetuximab, panitumumab, SYM004 and MM-151 (range, 0.01–25 μg/ml) for 96 hours. The proliferation rate was evaluated by MTT assay. The IC_50_ value was determined by interpolation with dose-response curves. Results represent the mean of three separate experiments, each performed in duplicate.

Figure S1


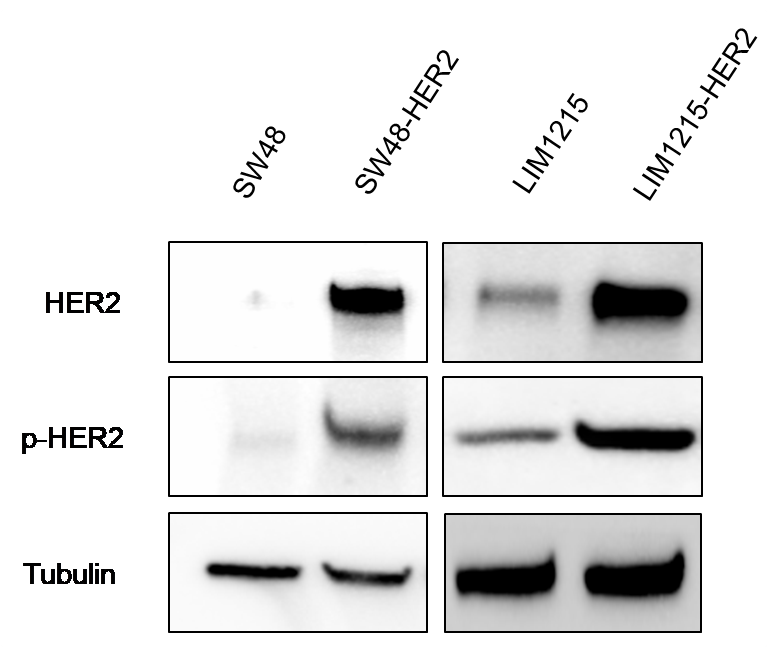


Figure S2


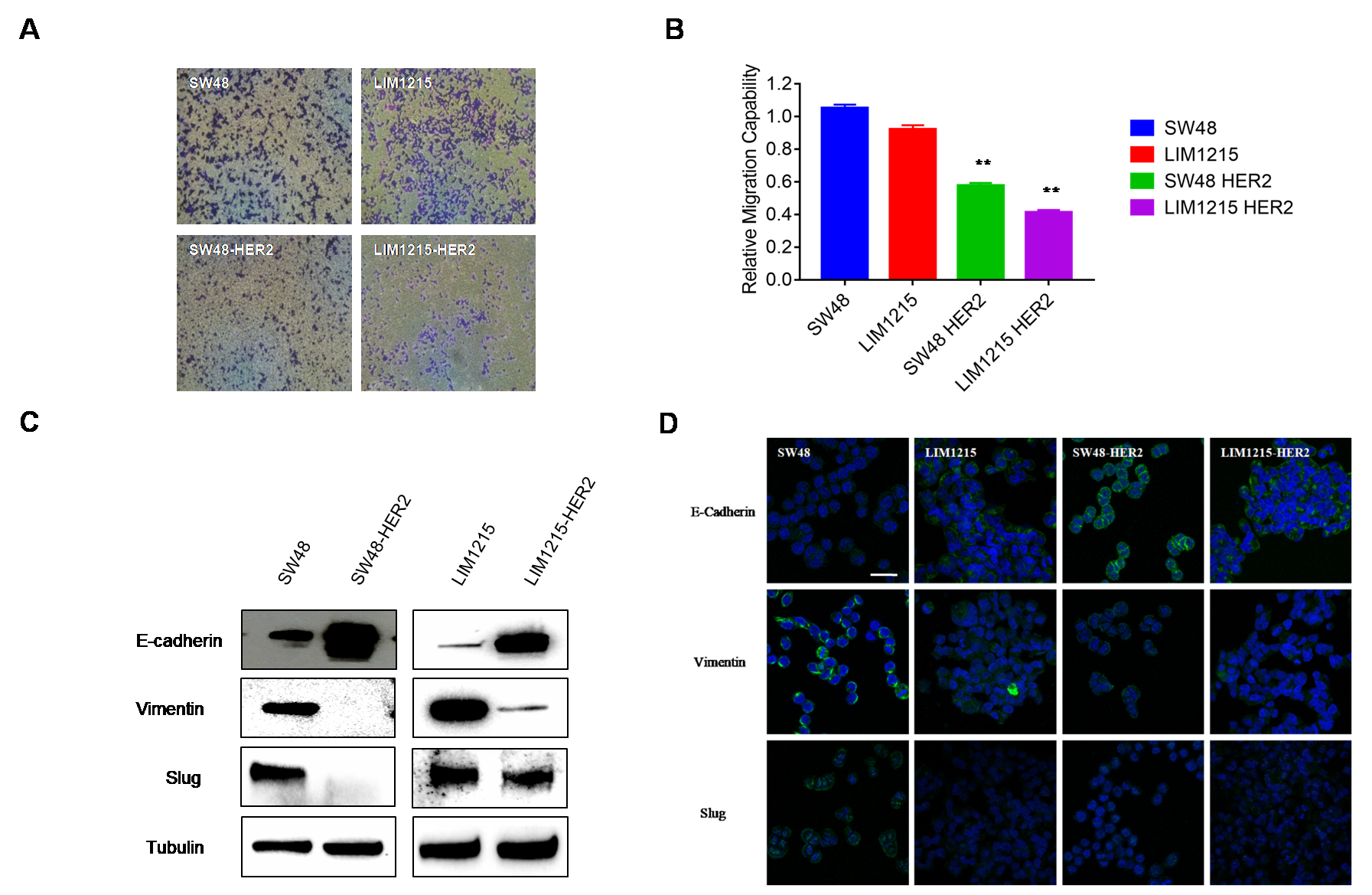


Figure S3


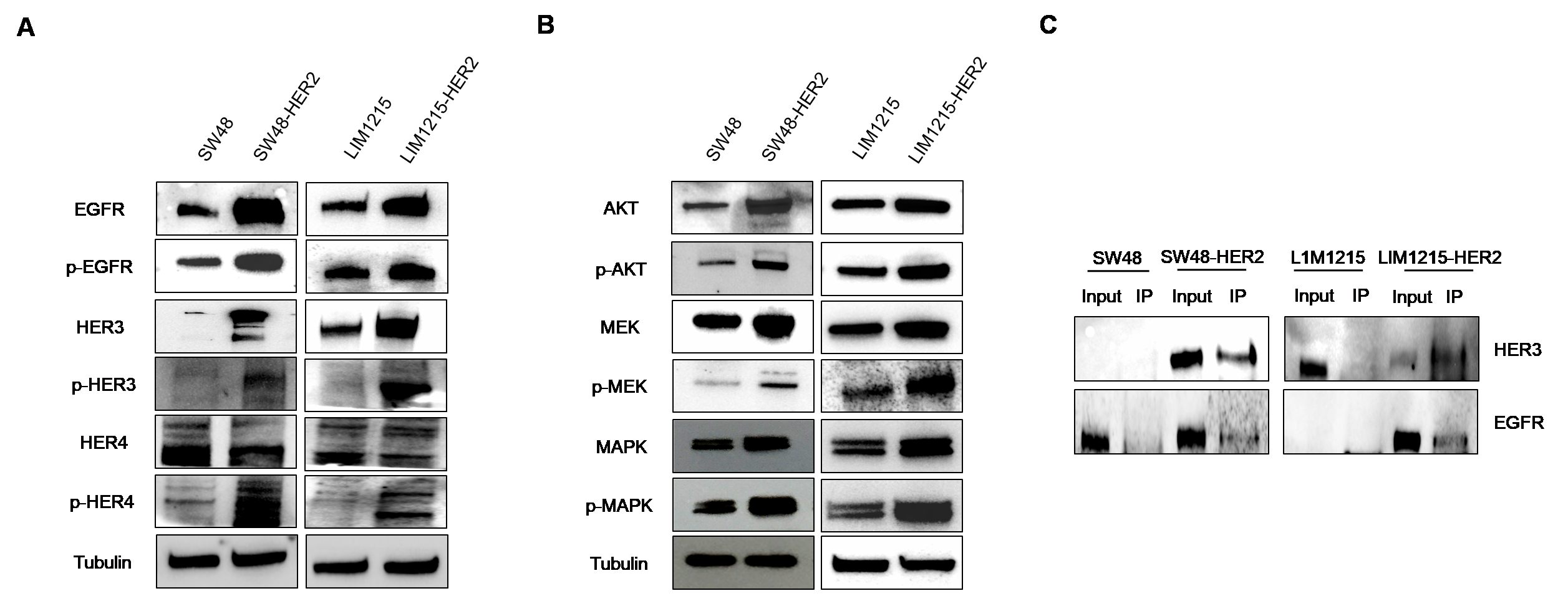


Figure S4


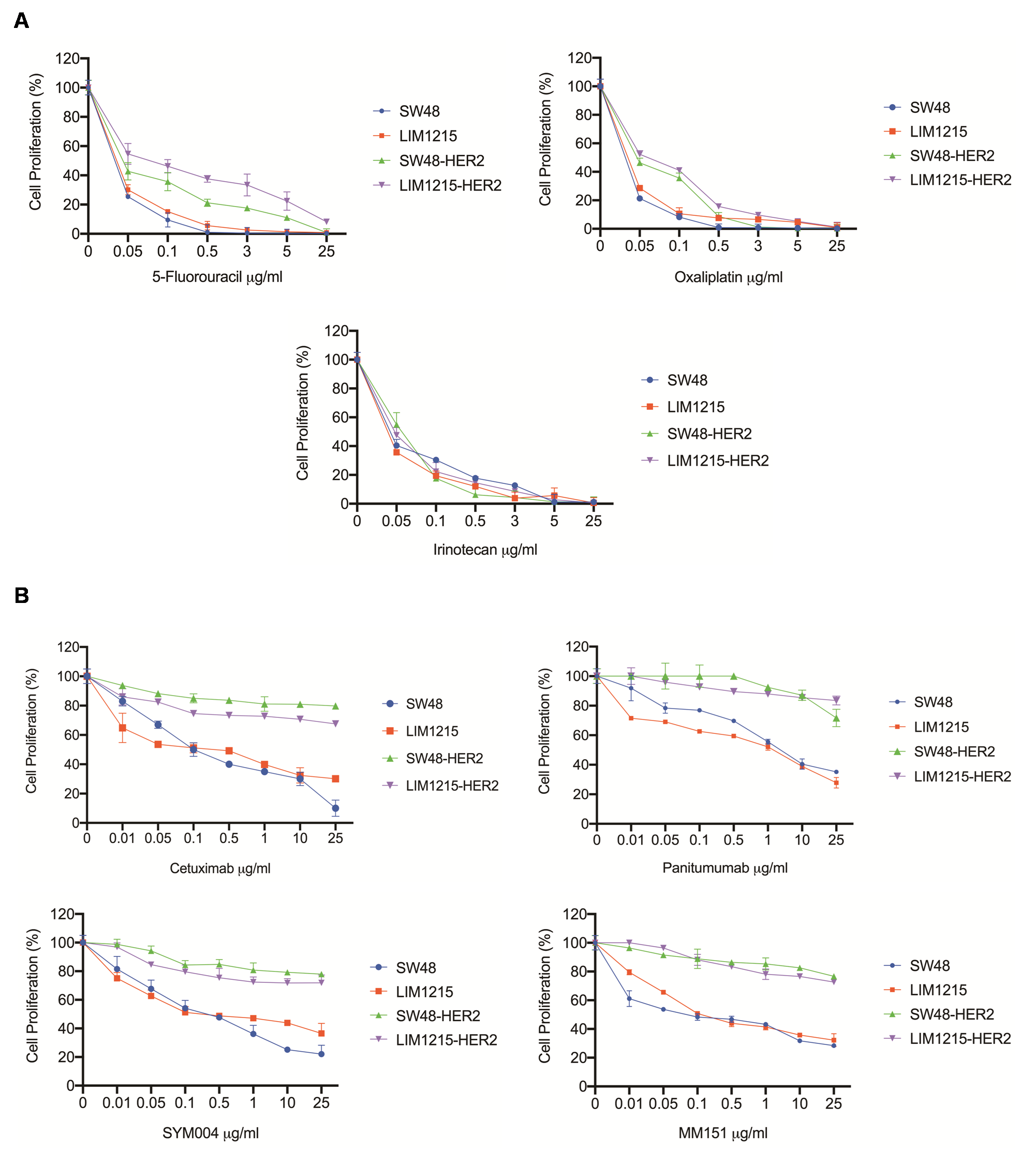

Supplement: Supplementary file 1 — Figure S1. Expression and phosphorylation of HER2 in parental SW48 and LIM1215 human colon cancer cell lines and in their HER2-amplified derivatives (SW48-HER2 and LIM1215-HER2) cells (Additional file 3: Supplementary Methods). Figure S2. Phenotypic characterization of parental SW48 and LIM1215 human colon cancer cell lines and of their HER2-amplified derivatives (SW48-HER2 and LIM1215-HER2) cells. Figure S3. Expression and phosphorylation of HER family receptors and their downstream signaling pathways in parental SW48 and LIM1215 human colon cancer cell lines and in their HER2-amplified derivatives (SW48-HER2 and LIM1215-HER2) cells. Figure S4. Effects of chemotherapeutic agents and of anti-EGFR monoclonal antibodies on cell proliferation in parental SW48 and LIM1215 human colon cancer cell lines and in their HER2-amplified derivatives (SW48-HER2 and LIM1215-HER2) cells. (DOCX 5258 kb) [file 13046_2019_1230_MOESM1_ESM.docx]
