# Supplementary material for: Combined blockade of MEK and PI3KCA as an effective antitumor strategy in HER2 gene amplified human colorectal cancer models
Source: J Exp Clin Cancer Res. 2019 Jun 4;38:236. doi: 10.1186/s13046-019-1230-z (PMC6549349; doi:10.1186/s13046-019-1230-z)
Supplement: Supplementary file 2 — Table S1. Evaluation of protein expression level in parental (SW48 and LIM1215) and in HER2-amplified human colon cancer cell lines. Legend: Negative symbol (-) no protein expression detected; Positive symbols: (+) expression and (++) over-expression levels of each protein detected. Table S2. Antitumor efficacy of oxaliplatin plus trastuzumab followed by maintenance treatment in human HER2-amplified colon cancer xenograft. (DOCX 613 kb) [file 13046_2019_1230_MOESM2_ESM.docx]

**Additional Table S1.** Evaluation of protein expression level in parental (SW48 and LIM1215) and in *HER2*-amplified human colon cancer cell lines. Legend: Negative symbol (-) no protein expression detected; Positive symbols: (+) expression and (++) over-expression levels of each protein detected.

**
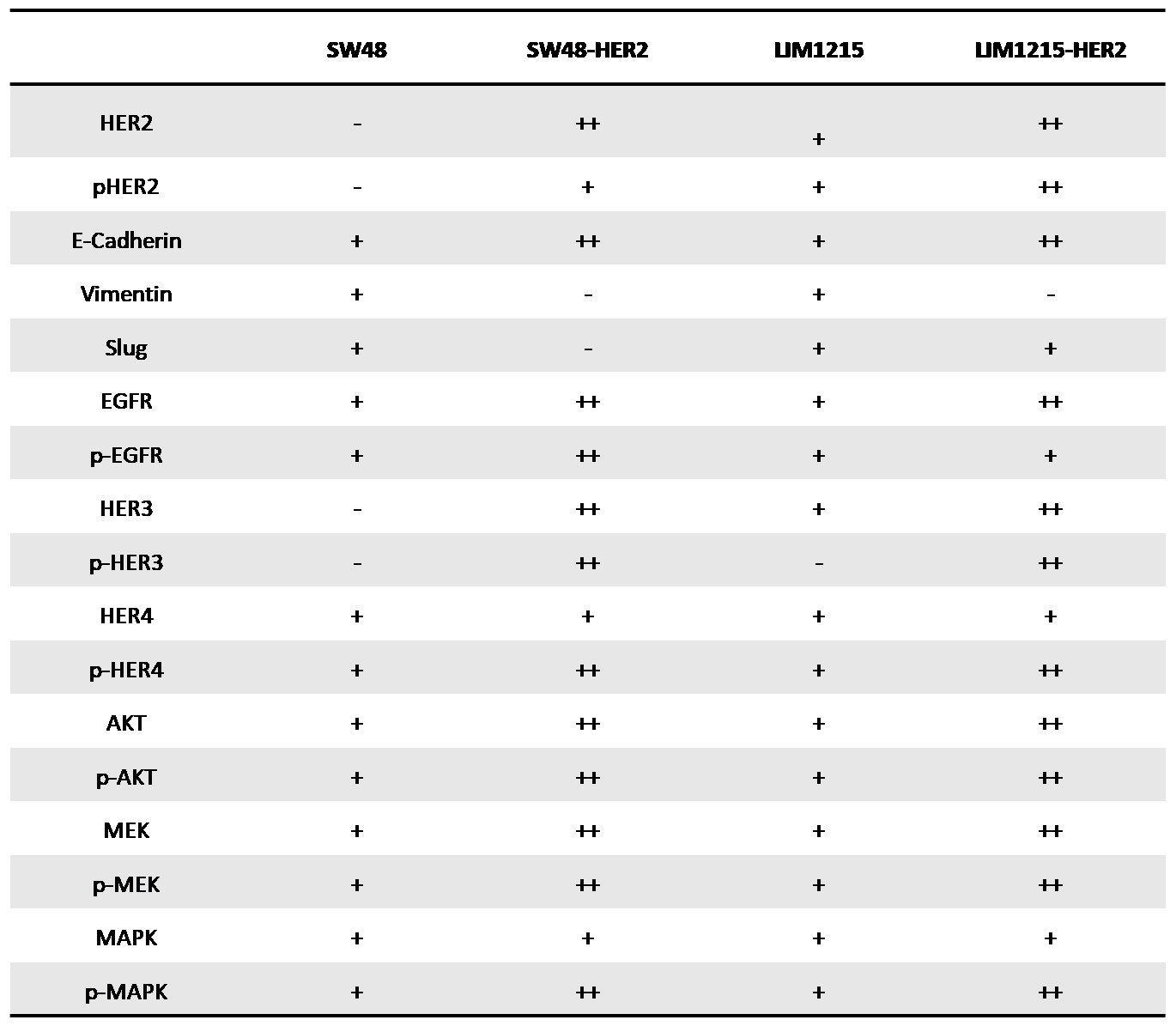
**

**Additional Table S2.** Antitumor efficacy of oxaliplatin plus trastuzumab followed by maintenance treatment in human HER2-amplified colon cancer xenograft.

A) LIM1215-HER2 amplified colon cancer xenograft


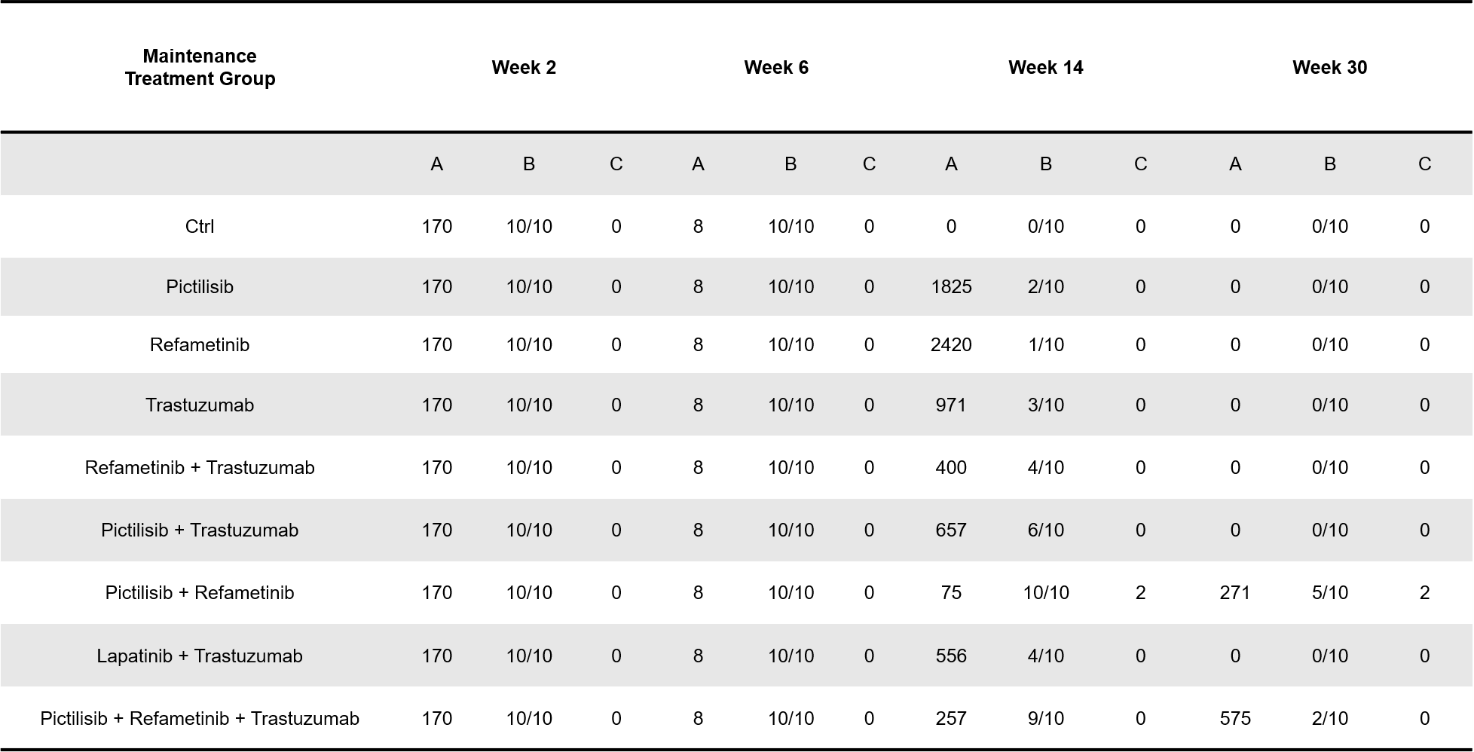


B) SW48-HER2 amplified colon cancer xenograft


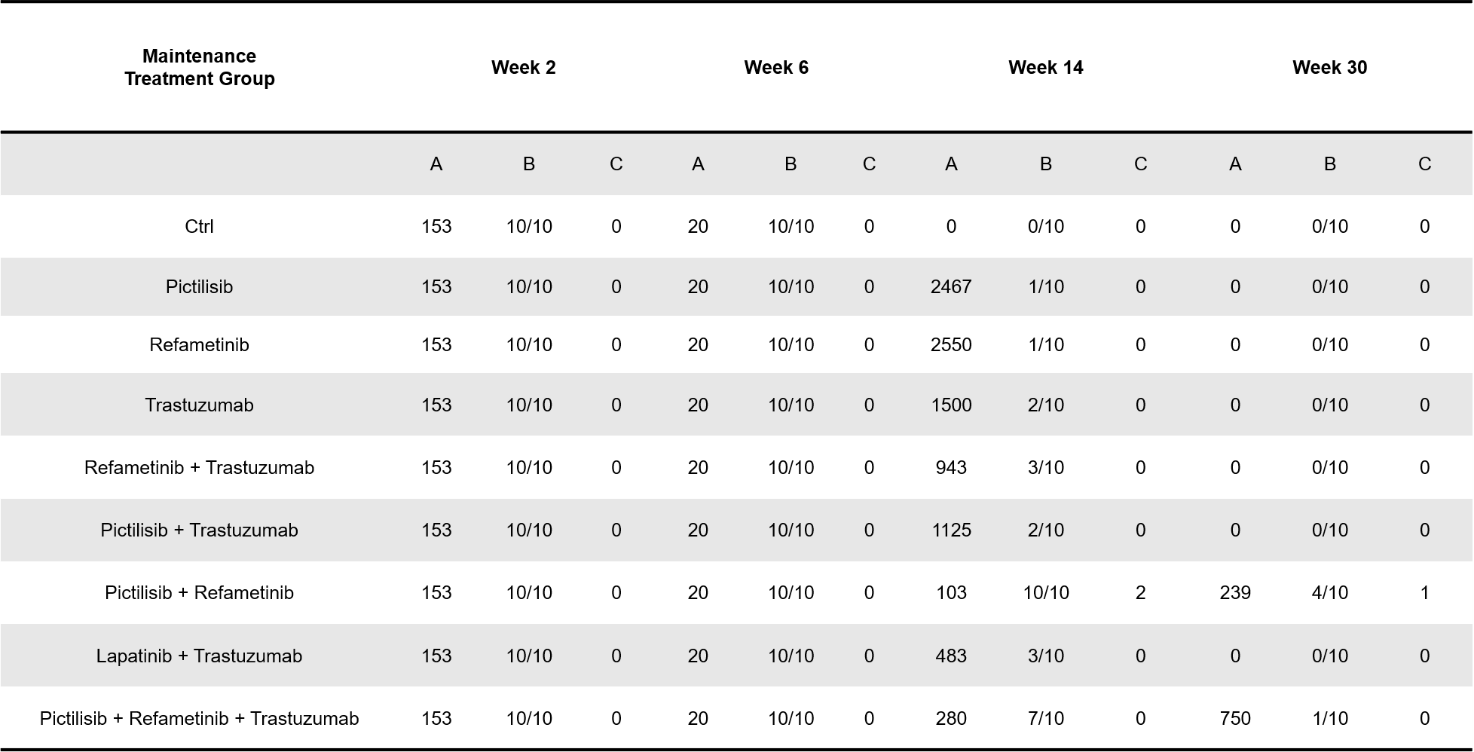


Mice injected subcutaneously with indicated colon cancer cell lines and treated with oxaliplatin plus trastuzumab from week 2 to week 6. Subsequently from week 6 to week 14 were randomly divided in nine groups and treated with indicated drugs. Tumor volume was measured three times *per* week until week 30. Animals were sacrificed when tumors achieved 2,500 mm^3^ in size. Values are expressed as mean for each group over the given time frame. Ctrl, control; A: median tumor volume (mm^3^); B: alive mice/total mice; C: number of mice with clinical complete remission.
