# Supplementary material for: Combined blockade of MEK and PI3KCA as an effective antitumor strategy in HER2 gene amplified human colorectal cancer models
Source: J Exp Clin Cancer Res. 2019 Jun 4;38:236. doi: 10.1186/s13046-019-1230-z (PMC6549349; doi:10.1186/s13046-019-1230-z)
Supplement: Supplementary file 3 — Supplementary Methods. (DOCX 18 kb) [file 13046_2019_1230_MOESM3_ESM.docx]

**Additional Methods**

**Transfection**

The SW48 and LIM1215 human colon cancer cell lines were transfected with 5 μg of pcDNA3 HER2 plasmid (Addgene) using FuGENE® HD Transfection Reagent (Promega) following manufacturer’s instructions, as previously described [34] to generate their *HER2*-amplified derivatives cells (SW48-HER2 and LIM1215-HER2). The day before transfection, cells were plated in 100 mm^3^ dishes at 40 % of confluence in RPMI 1460 supplemented with 10% FBS. After 48 hours Geneticin® (GIBCO by Life Technologies) at final concentration of 1.25 mg/ml was used to select Geneticin–resistant mass population.

**Migration assay**

The SW48, LIM1215, SW48-HER2 and LIM1215-HER2 cellswere plated into the top of polycarbonate transwell filter (24-well inserts; pore size, 8 µm; BD Bioscience) at the density of 5 × 10^5^ cells/well for migration assay. Cells were seeded on upper chambers in serum-free media, while medium containing 10% FBS was used as a chemotractant in the lower chambers. Cancer cells were allowed to migrate for 72 hours and cells at the bottom of the chamber were stained with crystal violet. The picture of each well was taken to the images of the cells with the 10X objective. The number of migrated cells was calculated after cell lysates and quantified by spectrophotometer. Three independent experiments were performed and the data were presented as mean ± standard deviation (SD).

**Confocal microscopy for immunofluorescence staining**

For immunofluorescent staining of epithelial-mesenchymal-transition (EMT) markers (E-cadherin, Vimentin and Slug), 4×10^4^ cells were seeded on glass for 24 hours. Briefly, cells were fixed with 4% paraformaldehyde for 20 min and permeabilized with 0.1% Triton X-100 (Sigma-Aldrich, USA) for 10 min before staining for each EMT marker. Afterwards, Alexa Fluor488 goat anti-rabbit secondary antibodies (Molecular Probes, Invitrogen) were used; cell nuclei were stained with 4,6-diamidino-2-phenylindole (DAPI, Sigma). Samples were observed by confocal microscope (Ziess) with a 40X water objective. Images were acquired with a 1024 × 1024 pixels resolution.

**Western Blot analysis**

The SW48, LIM1215, SW48-HER2 and LIM1215-HER2 cells were seeded into 100 mm^3^ petri dishes for 24 hoursand basal expression of HER family receptors and downstream signaling pathway was evaluated. Protein lysates containing comparable amounts of proteins, estimated by a modified Bradford assay, were separated by 4–15% gradient mini precast TGX gel and transferred to nitrocellulose membrane (BioRad). The membranes were incubated with following primary polyclonal antibodies for total and phosphorylated forms: EGFR and phospho-EGFR, HER2 and phospho-HER2, HER3 and phospho-HER3, HER4 and phospho-HER4, AKT and phosphor-AKT, MEK1/2 and phospho-MEK1/2 and p44/42MAPK and phospho-p44/42 purchased from Cell Signaling. Monoclonal anti- α-tubulin antibody was provided by Sigma-Aldrich. After incubation with secondary anti-goat antibody at room temperature for 1 hours, according to the manufacturer’s instruction, the membranes were developed using an enhanced chemiluminescence (ECL) detection system (BioRad). Each experiment was done in duplicate. For immunoprecipitation cell lysates were prepared as described above, incubated with Protein G sepharose (Roche Diagnostics) following the Manufacturer's instructions and then immuno-precipitated with the anti-HER2 antibody. Briefly, the immune-precipitates were subjected to electrophoresis on 7,5% SDS-polyacrylamide gels and proteins were transferred to nitrocellulose membranes. The filters were incubated with the anti-HER3 and anti-EGFR antibodies and then detected using the ECL detection system (BioRad). Each experiment was done in duplicate.

**Proliferation assay**

The SW48 and LIM1215 human colon cancer cell lines and in their *HER2*-amplified derivatives (SW48-HER2 and LIM1215-HER2) were seeded into 24-well plates at the density of 1×10^4^ cells/well and exposed to different concentrations of 5-fluorouracil, oxaliplatin, irinotecan (range, 0.05-25 μg/ml), cetuximab, panitumumab, SYM004 and MM151 (range, 0.01-25μg/ml) for 96 hours. The growth inhibition was assessed by 3-(4,5-dimethylthiazol-2-yl)-2,5-diphenyltetrazoliumbromide (MTT-Sigma-Aldrich) assay after 96 hours of incubation. Results represent the median of three separate experiments, each performed in duplicate
